# Supplementary material for: Spatial population genetics in heavily managed species: Separating patterns of historical translocation from contemporary gene flow in white‐tailed deer
Source: Evol Appl. 2021 May 4;14(6):1673–89. doi: 10.1111/eva.13233 (PMC8210790; doi:10.1111/eva.13233)

**Supplementary Figures and Tables**

**Table S1:** Numbers (=N) of Arkansas white-tailed deer genotyped across ~35,000 SNP loci. Samples were collected from 75 counties (=County) from 2016-2019. Code indicates standard 2-letter county abbreviation. Samples removed due to missing data are not included.

| **County** | **Code** | **N** | **County** | | **Code** | | **N** |
| --- | --- | --- | --- | --- | --- | --- | --- |
| Arkansas | AR | 11 | Lee | | LE | | 9 |
| Ashley | AS | 1 | Lincoln | | LI | | 8 |
| Baxter | BA | 19 | Little River | | LR | | 10 |
| Benton | BE | 19 | Logan | | LO | | 32 |
| Boone | BO | 34 | Lonoke | | LN | | 11 |
| Bradley | BR | 2 | Madison | | MA | | 39 |
| Calhoun | CA | 14 | Marion | | MR | | 39 |
| Carroll | CR | 50 | Miller | | MI | | 8 |
| Chicot | CH | 3 | Mississippi | | MS | | 2 |
| Clark | CL | 7 | Monroe | | MO | | 10 |
| Clay | CY | 15 | Montgomery | | MN | | 2 |
| Cleburne | CE | 8 | Nevada | | NE | | 13 |
| Cleveland | CV | 5 | Newton | | NW | | 140 |
| Columbia | CO | 4 | Ouachita | | OU | | 12 |
| Conway | CN | 11 | Perry | | PE | | 8 |
| Craighead | CG | 7 | Phillips | | PH | | 9 |
| Crawford | CW | 6 | Pike | | PI | | 8 |
| Crittenden | CT | 8 | Poinsett | | PO | | 10 |
| Cross | CS | 10 | Polk | | PL | | 3 |
| Dallas | DA | 7 | Pope | | PP | | 62 |
| Desha | DE | 11 | Prairie | | PR | | 10 |
| Drew | DR | 14 | Pulaski | | PU | | 11 |
| Faulkner | FA | 14 | Randolph | | RA | | 8 |
| Franklin | FR | 20 | Saline | | SA | | 11 |
| Fulton | FU | 9 | Scott | | SC | | 2 |
| Garland | GA | 14 | Searcy | | SE | | 32 |
| Grant | GR | 5 | Sebastian | | SB | | 17 |
| Greene | GE | 16 | Sevier | | SV | | 12 |
| Hempstead | HE | 9 | Sharp | | SH | | 16 |
| Hot Spring | HS | 3 | St. Francis | | SF | | 7 |
| Howard | HO | 11 | Stone | | ST | | 15 |
| Independence | IN | 7 | Union | | UN | | 6 |
| Izard | IZ | 6 | Van Buren | | VB | | 23 |
| Jackson | JA | 2 | Washington | | WA | | 17 |
| Jefferson | JE | 13 | White | | WH | | 10 |
| Johnson | JO | 45 | Woodruff | | WO | | 9 |
| Lafayette | LA | 14 | Yell | | YE | | 31 |
| Lawrence | LW | 17 | **Total** | |  | | **1,143** |
|  |  |  | |  |  |  | |

**Figure S1**: **Effective migration rates and intra-population diversity** (log_10_ scale) for Arkansas white-tailed deer, calculated from effective migration surfaces (EEMS). Rates are plotted according to colored bin, with divisions calculated as natural breaks using the Jenks algorithm in ArcMAP.


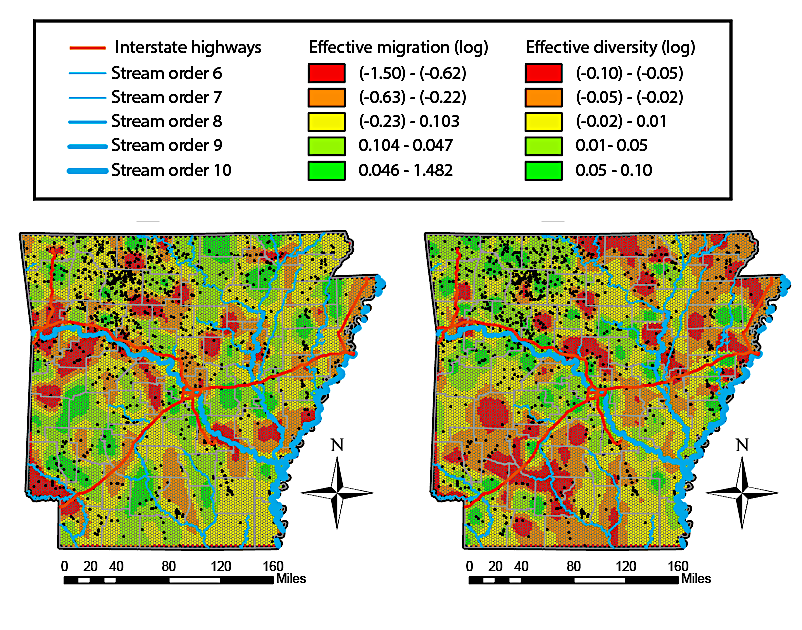


**Figure S2: Sampled transects employed for genomic cline analysis of Arkansas white-tailed deer,** with selected groups within each coalesced within colored hulls: Reference population 1 (p1; red); Reference population 2 (p2; green); and putative admixed individuals (popA; blue).


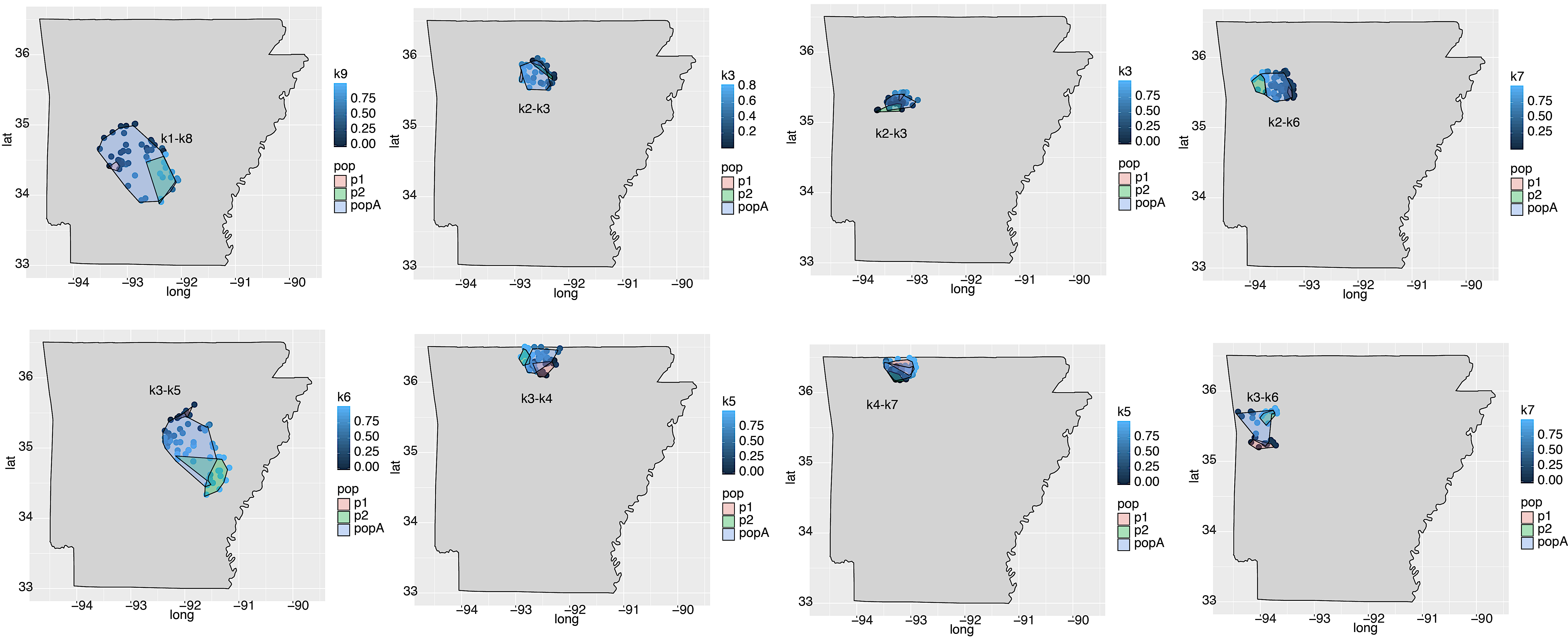


**Figure S3: LOCATOR Geo-located results for CWD-positive Arkansas white-tailed deer derived via LOCATOR.** Individuals are represented by dots, pairs of which are connected by a single line. A black dot denotes predicted location, while a colored dot indicates ‘true’ (observed = sampled) location (color proportional to distances separating each). Inserted figures offer full prediction results for three selected samples, with bootstrap estimates demarked by a 95% contour.


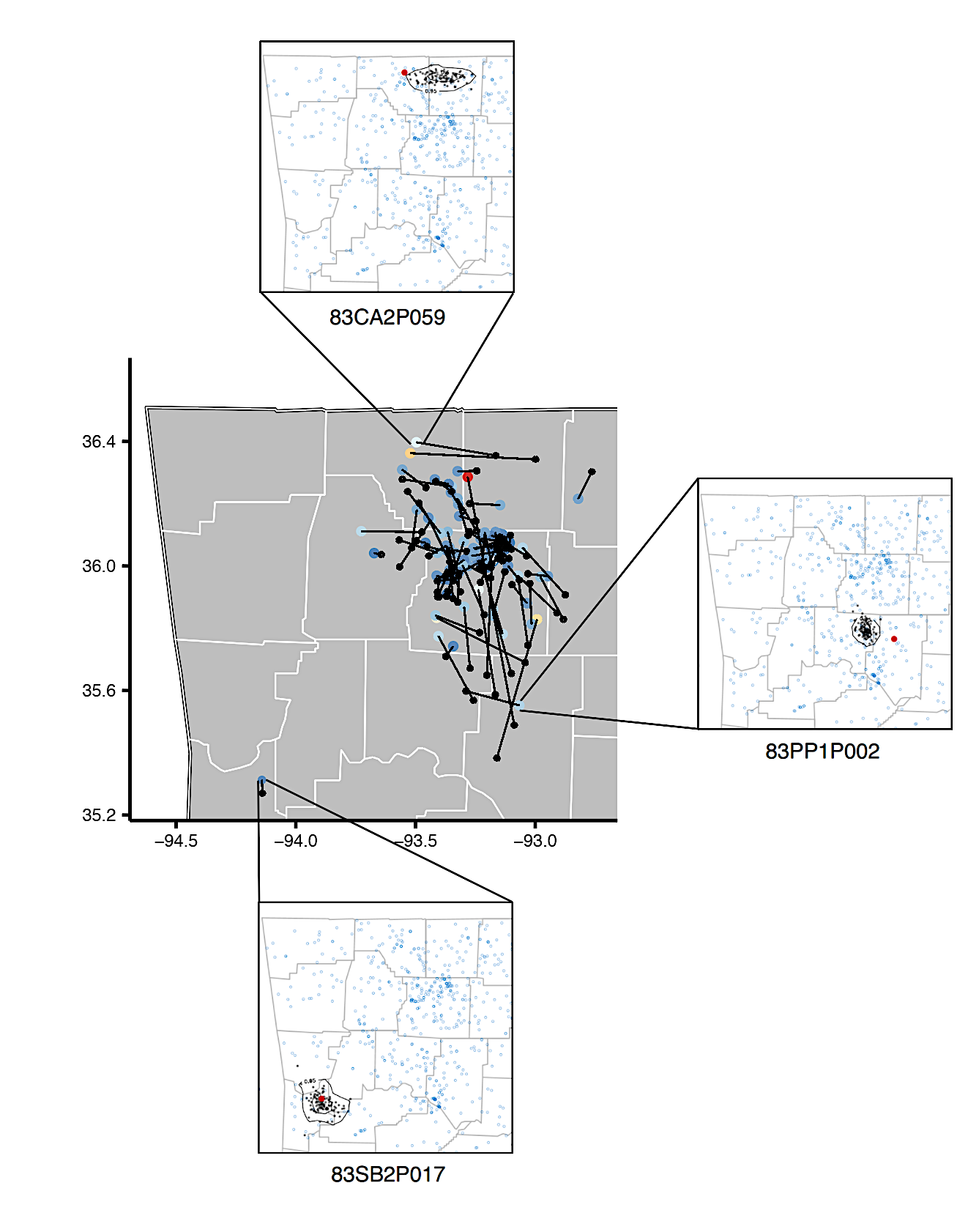


**Figure S4: Effects of error thresholds on inferred dispersal distances for Arkansas white-tailed deer, as predicted by LOCATOR analysis.** Inferred dispersal kernels were computed at several error thresholds: (A) Error computed as mean distance of predicted coordinates for each individual at 100 bootstrap estimates from predicted centroid (=interpretation of localized vs. dispersed predictions). Dispersal distances calculated as difference (in km) between predicted centroid versus ‘true’ (observed) location. Inferred dispersal distances generally increase for individuals with larger prediction error; (B) Larger stochastic variation in centroid location for individuals with lower predicted precision among bootstraps; (C) Standard deviation of dispersal distances for the latter computed in a sliding window of 10km along the x-axis (prediction error).


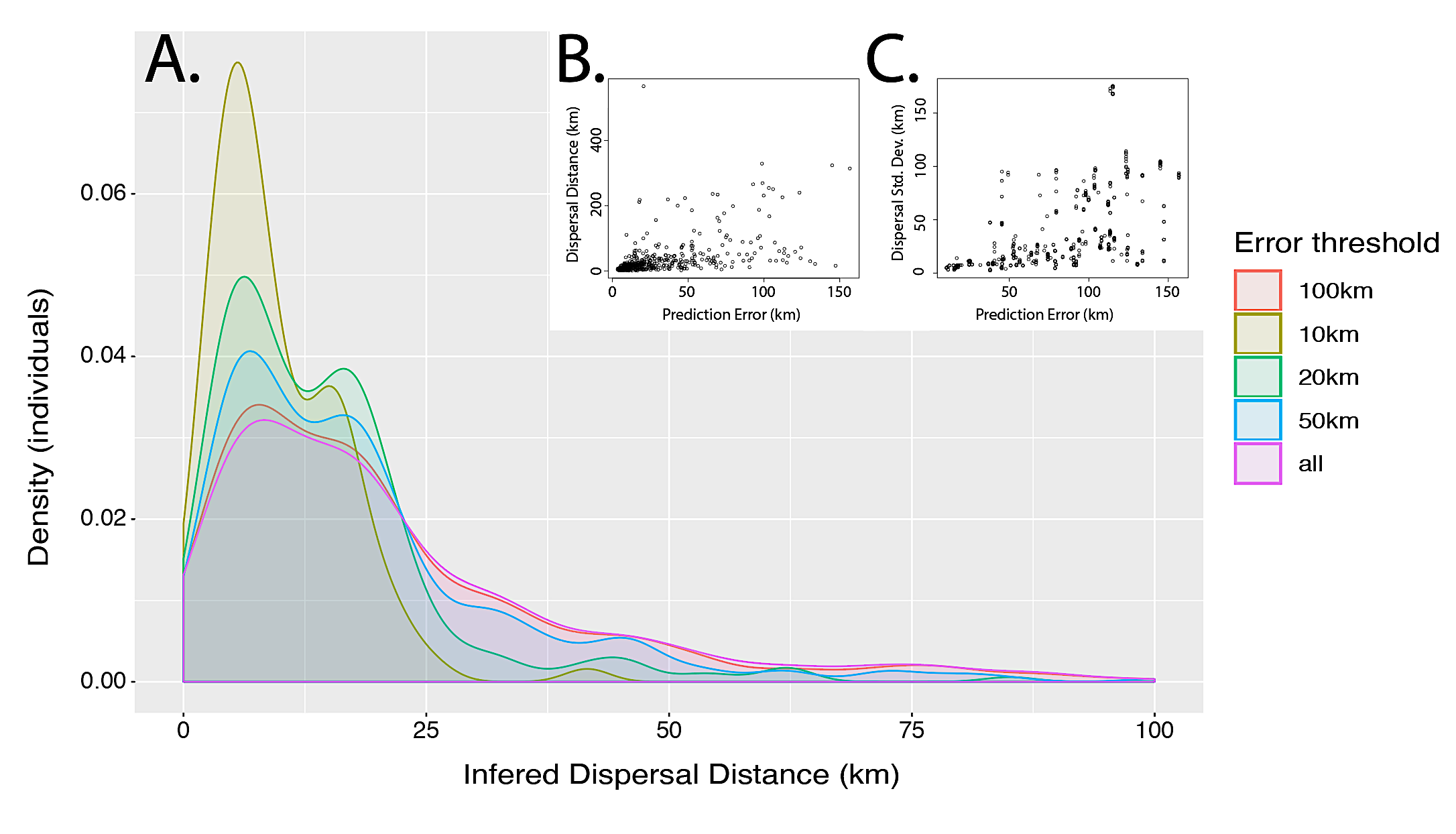


**Figure S5: Spatial patterns of genetic dissimilarity among Arkansas white-tailed deer partitioned by age and sex.** Genetic distances between individuals and neighbors derived from 5,000 randomly sampled SNPs depicted across physical distance (x-axis). Results depict different cohorts of males (A) and females (B) by age.


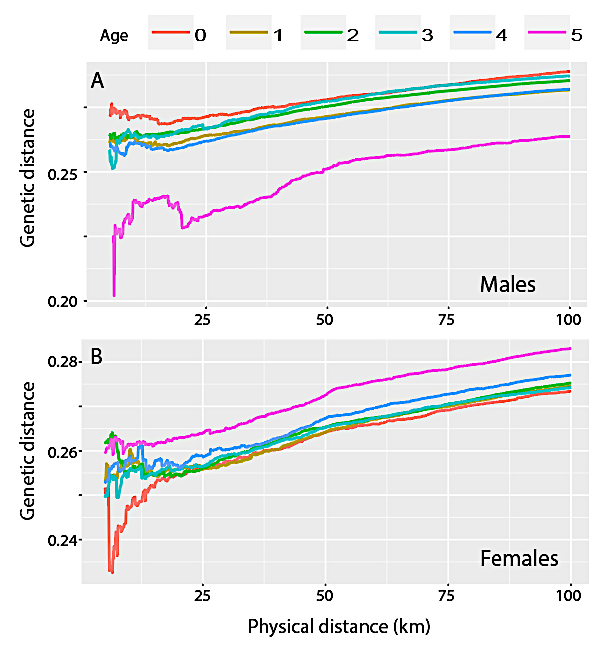


**Figure S6**: **Census estimates for Arkansas white-tailed deer from surveys in 1942-1946.**


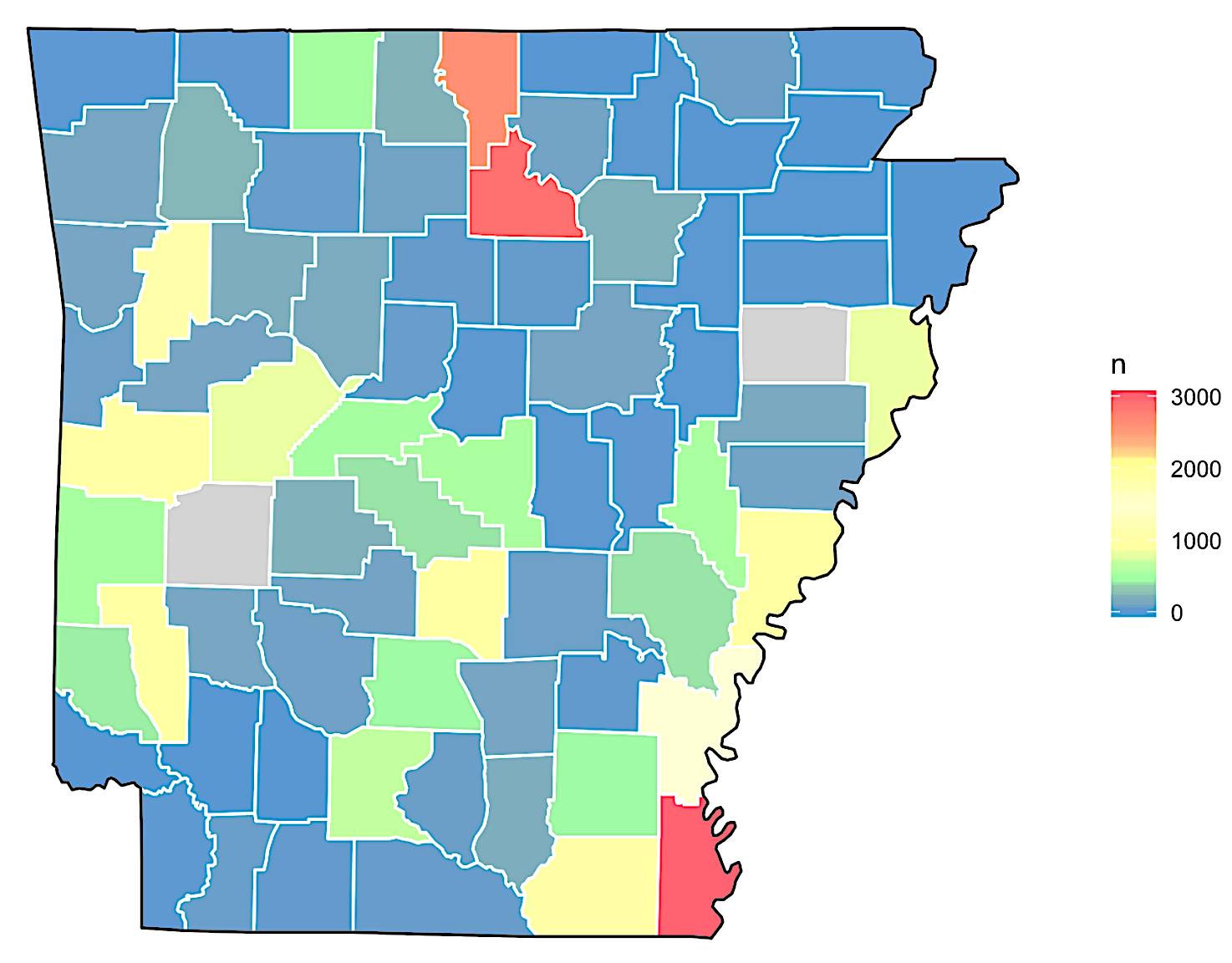


**Figure S7: Subpopulation probabilities overlain with ‘deer occupied territories’ in Arkansa circa 1942-1947** (in green). Polygons representing deer occupation were manually compiled from the Wildlife and Cover Map of Arkansas (1942-47), prepared by Arkansas Game and Fish Commission in cooperation with U. S. Fish and Wildlife Service as one aspect of a Federal Aid Project (drawn by Flaun M. Tolar). Map accessed from the University of Arkansas Library Arkansas Collection (Special Collections). A higher resolution geo-referenced version available at a later date (reproduction/ copy permissions granted by University of Arkansas Libraries).


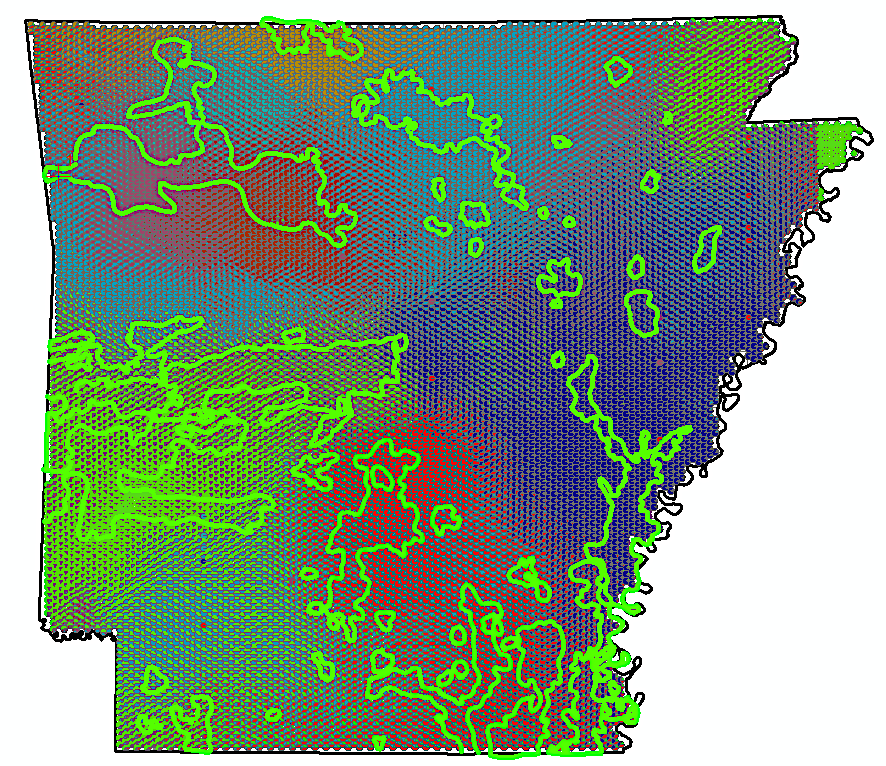

Supplement: Supplementary file 1 — Supplementary Material [file EVA-14-1673-s001.docx]
